# The Effect of Arabic Gum on Renal Function in Reversible Unilateral Ureteric Obstruction

**DOI:** 10.3390/biom9010025

**Published:** 2019-01-11

**Authors:** Fayez T. Hammad, Suhail Al Salam, Abderrahim Nemmar, Mahmoud Ali, Loay Lubbad

**Affiliations:** 1 Department of Surgery, College of Medicine & Health Sciences, United Arab Emirates University, Al Ain 17666, UAE; Loay_Lubbad@uaeu.ac.ae; 2 Department of Pathology, College of Medicine & Health Sciences, United Arab Emirates University, Al Ain 17666, UAE; suhaila@uaeu.ac.ae; 3 Department of Physiology, College of Medicine & Health Sciences, United Arab Emirates University, Al Ain 17666, UAE; anemmar@uaeu.ac.ae; 4 Department of Pharmacology, College of Medicine & Health Sciences, United Arab Emirates University, Al Ain 17666, UAE; malhaj_ali@uaeu.ac.ae

**Keywords:** Arabic gum, renal functions, ureteric obstruction, oxidative stress, histology

## Abstract

Arabic gum (AG) has antioxidant and anti-inflammatory properties. However, the effect of AG in ureteric obstruction (UO) has not been investigated yet. Male rats underwent reversible left unilateral UO (UUO) for 72 h. Group AG-1 (*n* = 12) received AG 15 g/kg/day dissolved in drinking water starting seven days before and continuing throughout the period of the UUO, whereas group Vx-1 (*n* = 8) had only water. Group AG-2 (*n* = 12) and Vx-2 (*n* = 8) had similar protocols as AG-1 and Vx-1, respectively, but underwent terminal experiments to measure renal functions, six days post-UUO reversal. Arabic gum significantly attenuated the UUO-induced increase in the tissue level of malonedialdehyde and superoxide dismutase and the rise in the gene expression of TNF-α, TGF-β1, and p53 in AG-1 compared to Vx-1. It also attenuated the severity of tubular dilatation. However, AG did not affect the alterations in the renal blood flow or glomerular filtration rate. The fractional sodium excretion was lower in AG-2 but did not reach statistical significance (0.40 ± 0.11 vs 0.74 ± 0.12, *p* = 0.07). AG attenuated the UUO-induced rise in oxidative stress markers and proinflammatory and profibrotic cytokines and the degree of renal tubular dilatation, indicating a protective effect in obstructive nephropathy.

## 1. Introduction

Ureteric obstruction (UO) is one of the common causes of renal injury, which might cause temporary or permanent renal impairment [1]. The renal damage is due to interactions of various factors and mediators at the time of obstruction leading to alterations in renal function [2,3,4]. Several drugs and agents were shown to ameliorate this damage through their effects on these mediators [2,5,6,7,8,9,10].

Recently, there has been a growing interest in using natural phytochemical compounds as treatment alternatives in several conditions including renal diseases [5,8,11,12,13,14]. This is due to their relatively low toxicity, low price, and wide availability. Moreover, in some cultures, it is more acceptable to use these compounds compared to conventional medications. Indeed, it has been estimated that at least 25% of the drugs used over the past few decades were directly derived from plants and another approximately 25% were chemically altered natural products [15].

Arabic gum (AG) is one of these natural compounds and is composed of water-soluble dietary fibers that are produced from the dried gummy exudates of the stems and branches of *Acacia senegal* and *Acacia seyal* [16]. It is a branched-chain, complex polysaccharide, either neutral or slightly acidic, and is found as a mixture of calcium, magnesium, and potassium salts of the polysaccharidic acid. The use of AG gum dates back to the ancient Egyptians who used it as an adhesive and ink stabilizer [17]. Currently, AG is widely used in industry. For instance, it is used as a thickening agent, stabilizer, and emulsifier in certain food industry products, such as soft drinks [18].

Historically, AG has been used as a folk medicine in some renal conditions, such as renal failure [19]. Experimentally, the effect of AG has been investigated in several diseases and conditions, such as hepatic [20], cardiac [21], and renal [22] toxicities and in diabetes mellitus [23]. A recent clinical trial has shown a protective effect of AG in patients with sickle cell anemia [24]. These protective effects of AG are likely to be related to its anti-oxidant and anti-inflammatory properties. Among several conditions, there is evidence to suggest that it has beneficial effects in acute and chronic renal failure of various etiologies [19,22,25]. However, the effect of AG on the renal dysfunction following UO has not been previously investigated. The aim of this study is to investigate this effect using a rat model of reversible unilateral ureteric obstruction (UUO) by assessing various physiological, biochemical, and histopathological effects of AG in this condition.

## 2. Methods

Studies were performed in male Wistar rats weighing 211–230 g at the time of UUO. Rats were housed in standard cages and kept in a 12-h light–dark cycle at 20 °C. Animals were fasted for 12 h before the experimental procedures but had water ad libitum. The protocol was approved by the local animal research ethics committee (#A [5–15]).

### 2.1. Ureteric Occlusion Operation and Reversal

Under aseptic conditions, animals were anesthetized with ketamine hydrochloride (80 mg/kg, intraperitoneally, Pantex Holland B.V., Glendenning, NSW Holland) and xylazine hydrochloride (8 mg/kg, intraperitoneally, Troy Laboratory PTY Limited, Glendenning, NSW, Australia). Following exposure of the left ureter through a midline abdominal incision, 3–4 mm of bisected PVC tubing (0.58 mm internal diameter) was placed around the middle part of the left ureter and the tubing was constricted with a 4-0 silk suture to occlude the ureter. This was followed by wound closure.

Reversal of the UO was performed 72 h later through the same incision using a dissecting microscope. The obstructing tube was removed, and full release of the obstruction was confirmed by observing a free flow of urine across the site of obstruction.

### 2.2. Arabic Gum Administration and Experimental Groups

**Figure 1:**
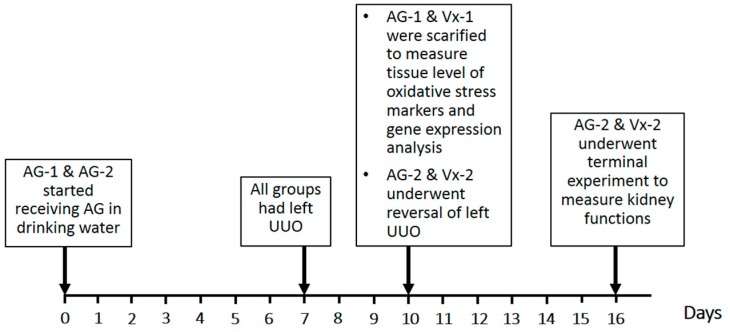
Schematic presentation of the study plan showing interventions in all groups. AG is Arabic gum, UO is ureteric obstruction, UUO is unilateral UO.

Arabic gum (Sigma-Aldrich, St. Louis, MO, USA) was administered at a dose of 15 g/kg/day dissolved in drinking water. There were four groups as shown in Figure 1:

1. AG-1 (*n* = 8) received AG starting 7 days prior to and continuing throughout the period of UUO but was sacrificed at the end of the 3 day period of obstruction to measure the tissue level of oxidative stress markers and gene expression of cytokines.
2. AG-2 (*n* = 12) received AG starting 7 days prior to and continuing throughout the period of obstruction until the terminal experiment six days post-UUO reversal.
3. Vx-1 (*n* = 8) underwent a similar protocol as AG-1 but no AG was added to the drinking water.
4. Vx-2 (*n* = 12) underwent a similar protocol as AG-2 but no AG was added to the drinking water.

### 2.3. Surgical Procedure in the Terminal Experiment and Measurement of Renal Functions

Both AG-2 and Vx-2 underwent terminal experiments six days following reversal of the UUO to measure renal functions. The surgical procedure was as previously described [26]. In summary, after anesthetizing the rats with pentobarbital sodium BP (60 mg/kg, intraperitoneally; J M Loveridge plc, Andover Hants SP10 5LQ, UK), the trachea and a femoral vein were cannulated. A femoral artery was then cannulated, and the tip of the cannula was positioned just below the level of the left renal artery to measure blood pressure. Through a midline abdominal incision, both kidneys were exposed, and their upper ureters were cannulated with polyethylene tubing (PE-10) for urine collection. Following surgery, a sustaining infusion of 0.9% saline with inulin (1.5% *w*/*v*) and para-aminohippuric acid (PAH) (0.2% *w*/*v*) was commenced. After one hour of equilibration, the experimental protocol consisted of two 20-min clearance periods to measure renal functions.

The value of glomerular filtration rate (GFR), renal blood flow (RBF), urine volume (UV), urinary sodium (U_Na_V), and fractional excretion of sodium (FE_Na_) was calculated as the average of the two clearance periods.

### 2.4. Gene Expression Analysis

Following the terminal experiment, the kidneys were removed and the middle part of each kidney was immediately excised, snap-frozen in liquid nitrogen, and stored at −80 °C for measurement of the gene expressions of the proinflammatory cytokine tumor necrosis factor-α (TNF-α), profibrotic cytokine transforming growth factor-β1 (TGF-β1), and the proapoptotic gene p53 as previously described [26].

In summary, total RNA was extracted using TRI Reagent^®^ Solution (Life Technologies Corporation, Grand Island, NY, USA). Quantity and quality of the extracted RNA were estimated using a NanoDrop instrument (Thermo Fisher Scientific Inc., Waltham, DE, USA). First-strand cDNAs were prepared in duplicate from the extracted RNA with GoScript™ Reverse Transcriptase (Promega Corporation, Madison, WI, USA) in the presence of RNasin^®^ Plus RNase inhibitor (Promega Corporation, WI, USA) and used as a template for gene expression analysis by real time PCR using TaqMan^®^ chemistry on Applied Biosystems^®^ 7500 Real-Time PCR instrument (Applied Biosystems, Foster City CA, USA). The reaction mixture consisted of 75 ng cDNA, TaqMan^®^ Universal Master Mix (Applied Biosystems, CA, USA), 0.6 μM of forward and reverse primers, and 0.25 μM of the fluorescent probes (Biosearch Technologies, Inc., Novato, CA, USA). Sequences of primers and fluorogenic probes are shown in Table 1. Primers and probes were designed using the online RealTimeDesign™ software (Biosearch Technologies) so that at least one of the primers was spanning an exon–exon junction within their respective gene. Ribosomal protein lateral stalk subunit P0 Rplp0 (coding for 60S acidic ribosomal protein P0) was used as the endogenous control gene for normalization between samples.

Changes in gene expression of target genes were estimated from calculated CT values using the Δ–Δ CT formula.

The results were expressed as the mean fold change of gene expression in the left obstructed kidney in the AG-1 and AG-2 compared to Vx-1 and Vx-2, respectively.

### 2.5. Measurement of Oxidative Stress Markers

The remaining part of the kidneys was rinsed with ice-cold PBS (pH 7.4) and homogenized in 0.1 M phosphate buffer (0.15 M KCl, 0.1 mM EDTA, and 0.1 mM phenylmethylsulfonylfluoride) at 4 °C. Cellular debris was removed from the homogenates by centrifugation for 10 min at 3000× *g* at 4 °C and supernatants were used for analysis. Protein content was measured by Bradford’s method as described previously [27].

NADPH-dependent membrane lipid peroxidation was measured as thiobarbituric acid reactive substance using malonedialdehyde (MDA) as standard (Sigma-Aldrich Fine Chemicals, St Louis, MO, USA). Measurement of reduced glutathione (GSH) and superoxide dismutase (SOD) levels were carried out using commercially available kits (Sigma-Aldrich Fine Chemicals, Germany and Cayman Chemical, Ann Arbor, MI, USA, respectively).

### 2.6. Histological Studies

The kidney tissue was washed with ice-cold saline and blotted with filter paper. The kidney tissue was then cassetted and fixed directly in 10% neutral formalin for 24 h, which was followed by dehydration in increasing concentrations of ethanol, clearing with xylene, and embedding with paraffin. Three-μm sections were prepared from paraffin blocks and stained with hematoxylin and eosin. The stained sections were evaluated using light microscopy in a blind fashion. The percentage of dilated tubules per low power field (10× area (each low power area equals to 2 mm^2^) in the cortex and medulla was determined with the help of Image J software (NIH, USA).

### 2.7. Statistical Analysis

Statistical analysis was performed using SPSS V16.0. Results are expressed as means ± SEM. Comparison between groups was carried out using one-way factorial ANOVA and comparison between the right control and the left obstructed kidney in each group was performed by the unpaired *t*-test. P values of less than 0.05 were considered statistically significant.

## 3. Results

### 3.1. Oxidative Stress Markers

**Figure 2:**
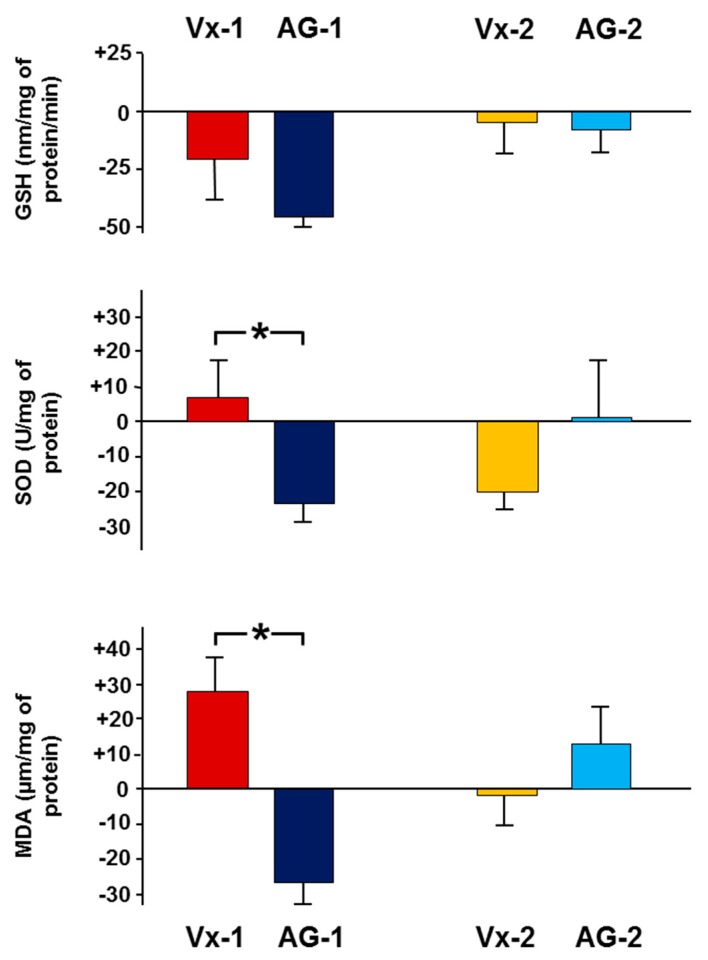
The percentage difference in the tissue concentration of malonedialdehyde (MDA), reduced glutathione (GSH), and superoxide dismutase (SOD) between the left and right kidneys in all groups. Values represent mean ± SEM. * indicates statistical significance between corresponding groups.

Figure 2 shows the percentage difference in the tissue level of oxidative stress markers between the left obstructed and the right control kidneys in all groups. When AG-1 was compared to Vx-1, there was a decrease by 27.2 ± 6.0 in the level of MDA in the AG-1 compared to an increase by 28.4 ± 9.0 in Vx-1 (*p* = 0.002). Similarly, there was a significant decrease in the SOD (−23.3 ± 5.0 vs 7.2 ± 9.2, *p* = 0.049). However, there was no significant difference in GSH level (−44.8 ± 4.6 vs −21.1 ± 19.3, *p* > 0.05). Six days post-reversal, there was no difference in the percentage difference between the left and right kidneys between AG-2 and Vx-2 (MDA: 14.2 ± 10.3 vs −1.2 ± 10.6, SOD: 0.8 ± 18.2 vs −19.5 ± 7.0, GSH: −6.8 ± 10.6 4 vs −4.1 ± 13.5, *p* > 0.05 for all).

### 3.2. Gene Expression Analysis Results

**Figure 3:**
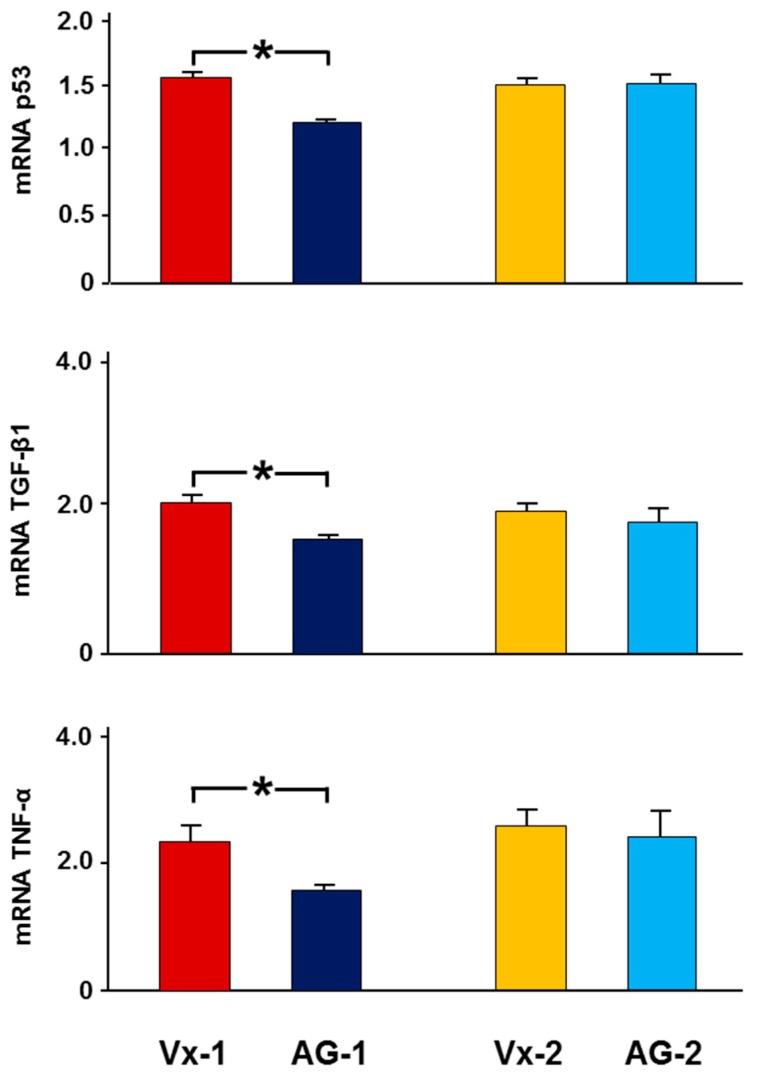
The expression of TNF-α, TGF-β1, and p53 in all groups. The results are expressed as the mean fold changes of gene expression in the left kidney compared to right kidney. Values represent mean ± SEM. * indicates statistical significance between corresponding groups.

As demonstrated in Figure 3, in Vx-1, there was a 2.3 ± 0.3 fold increase in the expression of TNF-α in the left obstructed kidney compared to the right control kidney, whereas in AG-1, there was only a 1.6 ± 0.1 fold increase (*p* = 0.048). Similarly, the left to right kidney expressions of TGF-β1 and p53 were lower in AG-1 (1.7 ± 0.1 vs 2.0 ± 0.1, *p* = 0.05 and 1.25 ± 0.04 vs 1.56 ± 0.04, *p* = 0.039, respectively). Six days post-reversal, there was no difference in the gene expression of these cytokines in the left kidney between AG-2 and Vx-2.

### 3.3. Glomerular and Tubular Functions in Vx-2 and AG-2

The mean arterial blood pressure and heart rate in AG-2 and Vx-2 were similar (116 ± 2 vs 114 ± 4 and 439 ± 8 vs 442 ± 9, *p* > 0.05 for both).

**Figure 4:**
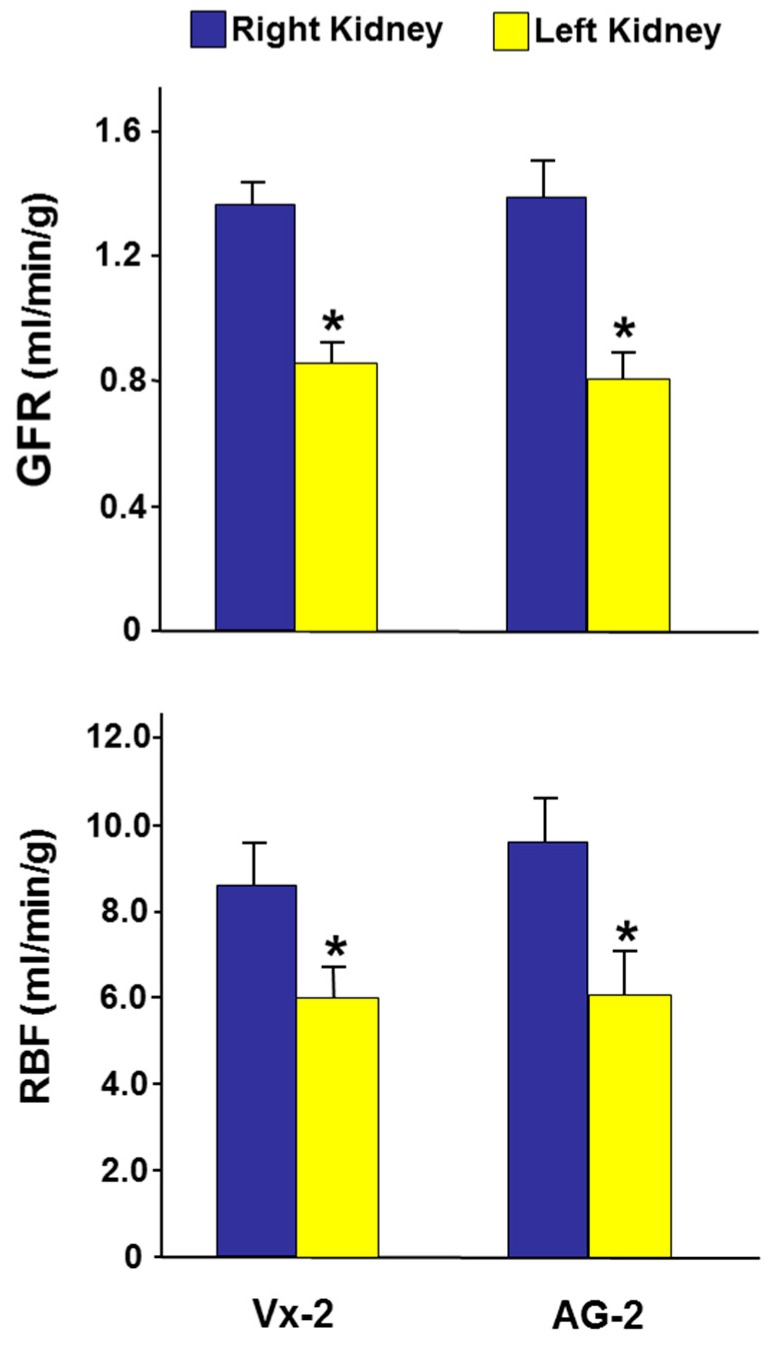
The glomerular filtration rate (GFR) and renal blood flow (RBF) in the right and left kidneys in Vx-2 and AG-2 six days following reversal of left ureteric obstruction. Values represent mean ± SEM. * indicates statistical significance between the right and left kidney within the same group.

**Figure 5:**
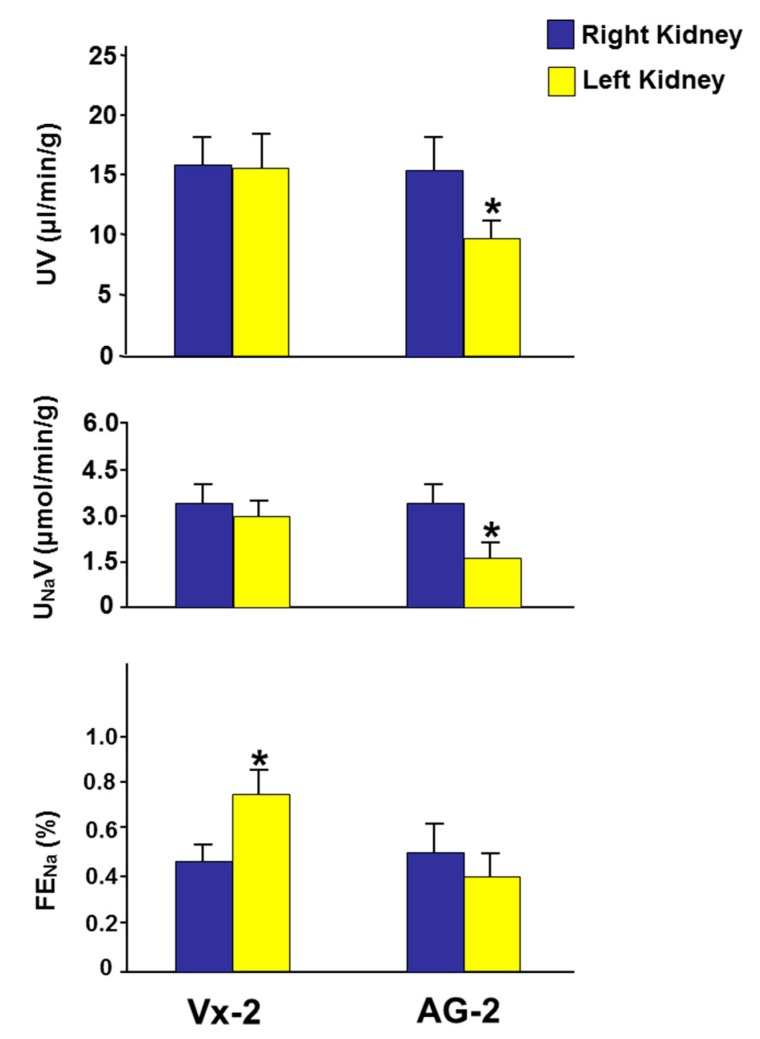
The tubular functional parameters including urine volume (UV), urinary sodium (U_Na_V), and fractional excretion of sodium (FE_Na_) in both kidneys in Vx-2 and AG-1, six days following reversal of left ureteric obstruction. Values represent mean ± SEM. * indicates statistical significance between the right and left kidney within the same group.

As shown in Figure 4, in Vx-2 the left RBF, six days post-UUO reversal, was 69% of the right RBF (5.98 ± 0.75 vs 8.62 ± 0.90, *p* = 0.03). The left GFR was 63% that of the right GFR (0.86 ± 0.07 vs 1.36 ± 0.08, *p* = 0.001). With the decrease in both RBF and GFR, the fractional excretion of sodium (FE_Na_) was higher in the left obstructed kidney (0.74 ± 0.12 vs 0.46 ± 0.07, *p* = 0.048) (Figure 5). However, there were no differences in the urine volume (UV) and urinary sodium excretion (U_Na_V) between the left and right kidneys (15.8 ± 2.3 vs 16.2 ± 2.6 and 2.98 ± 0.51 vs 3.26 ± 0.70, *p* > 0.05 for both (Figure 5).

In AG-2, the left RBF was 61% of the right RBF (6.01 ± 1.07 vs 9.74 ± 1.08, *p* = 0.01). Similarly, the left renal GFR was 58% of the right GFR (0.80 ± 0.10 vs 1.39 ± 0.13, *p* = 0.001) (Figure 4). As shown in Figure 5, the FE_Na_ of the left kidney was not different from the right control kidney (0.40 ± 0.11 vs 0.51 ± 0.11 (*p* > 0.05). However, the UV and U_Na_V of the left kidney were lower than those of the right kidney (9.6 ± 2.5 vs 15.5 ± 2.4, *p* = 0.049 and 1.63 ± 0.57 vs 3.38 ± 0.63, *p* = 0.043, respectively).

When AG-2 was compared to Vx-2, all variables in the right non-obstructed kidneys were similar (*p* > 0.05 for all variables). When the left obstructed kidney was compared between the two groups, the RBF and GFR were similar in the two groups (Figure 4). However, the FE_Na_, U_Na_V, and UV of the left kidney were lower in AG-2 but this did not reach statistical significance (0.40 ± 0.11 vs 0.74 ± 0.12, *p* = 0.07; 9.6±2.5 vs 15.8 ± 2.3, *p* = 0.09; 1.63 ± 0.57 vs 2.98 ± 0.51, *p* = 0.09, respectively) (Figure 5).

### 3.4. Histological Studies

**Figure 6:**
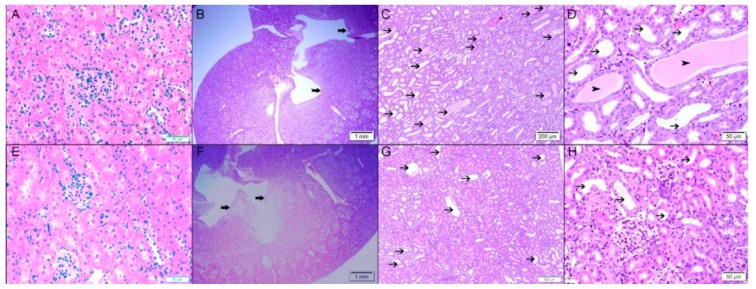
Histological features in the right nonobstructed and left obstructed kidneys after three days of left UUO in both Vx-1 and AG-1. Upper panel (Vx-1): (**A**), right kidney: normal kidney architecture and histology with no dilated tubules and normal calices; (**B**), left kidney with large areas of tubular dilatation in cortex, medulla, collecting ducts in renal papillary area, and dilated calices (thick arrow); (**C**,**D**), large areas of dilated tubules in cortex and medulla (thin arrow) with protein secretion in some of them (arrow head). Lower panel (AG-1): (**E**), right kidney: normal kidney architecture and histology with no dilated tubules and normal calices; (**F**), left kidney showing variable areas of tubular dilatation in cortex, medulla, collecting ducts in renal papillary area with dilated calices (thick arrow). (**G**,**H**), showing areas of dilated tubules in cortex and medulla (thin arrow) but less marked than the AG-1.

**Figure 7:**
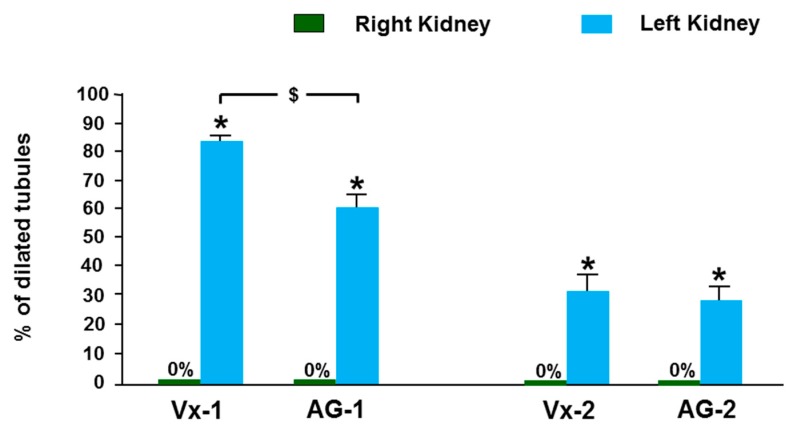
The degree of renal tubular dilatation in the right and left kidneys in all groups expressed as percentage. Values represent mean ± SEM. * indicates statistical significance between the right and left kidney within the same group.

As demonstrated in Figure 6, three days after the creation of the obstruction, the control right kidney in Vx-1 and AG-1 showed normal kidney architecture and histology with 0% dilated tubules (Figure 7). In Vx-1, the left obstructed kidney showed collecting duct dilation in the renal papillae with dilated calices and mild infiltration of renal papillae interstitial tissue with inflammatory cells, mainly lymphocytes. The cortical and superficial medullary tissue showed large areas of tubular dilatation and the degree of tubular dilatation in this group was 82.9 ± 3.3% (Figure 7). In AG-1, the left obstructed kidney showed similar histological features to the left kidney in Vx-1, including the inflammatory cell infiltrate and extracellular matrix, but there was a significantly lower degree of tubular dilatation (59.9 ± 3.57%, *p* = 0.0003).

**Figure 8:**
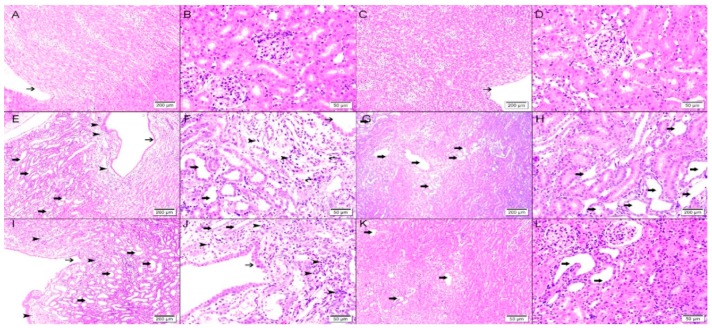
Histological features in the right non-obstructed and left obstructed kidneys six days post-UUO reversal in both Vx-2 and AG-2. Upper panel (right nonobstructed kidneys): (**A**,**B**), right kidney in Vx-2 showing normal kidney architecture and histology with no dilated tubules with normal calices (thin arrow); (**C**,**D**), right kidney in AG-2 with normal kidney architecture and histology and no dilated tubules with normal calices (thin arrow). Middle panel (left kidney in Vx-2): (**E**,**F**), shows collecting ducts dilation (thin arrow) in renal papillary area with dilated calices (thick arrow) with mild inflammatory cells infiltration of the renal papillae interstitial tissue consisting of lymphocytes mainly (arrow head); (**G**,**H**), the cortical and superficial medullary tissues show focal tubular dilatation (thick arrow). Lower panel (left kidney in AG-2): (**I**,**J**) shows collecting ducts dilation (thin arrow) in renal papillary area with dilated calices (thick arrow) with mild inflammatory cells infiltration of the renal papillae interstitial tissue consisting of lymphocytes mainly (arrow head); (**K**,**L**), the cortical and superficial medullary tissue show focal tubular dilatation (thick arrow).

Six days post-reversal of the UUO, the left previously obstructed kidney in Vx-2 had similar findings to the left kidney in Vx-1 but the findings were less marked (Figure 8) and the overall percentage of tubular dilatation in this group was 29.3 ± 6.7% (*p* < 0.0001) compared to Vx-1 (Figure 7). In AG-2, the left previously obstructed kidney showed similar histological features to the left kidney in Vx-2 (Figure 8) and the overall percentage of tubular dilatation in this group was 26.1 ± 6.3% (*p* > 0.05) compared to Vx-2.

## 4. Discussion

In this model of a three day reversible UUO, we have shown that the administration of AG prior to and during obstruction resulted in attenuation of the early changes in oxidative stress markers and in a significant early attenuation in the gene expression of some of the biomarkers of inflammation, fibrosis, and apoptosis, namely TNF-α, TGF-β1, and p53. Arabic gum also significantly attenuated some of the UUO-induced histological changes. This protective effect was accompanied by a tendency to improve renal tubular functional parameters.

The current study indicates that AG attenuated the UUO-induced increase in some of the oxidative stress markers. Previous studies reported an increase in MDA following UUO in rats [28,29], similar to the findings in this study. This early increase in the MDA concentration in the obstructed kidney compared with non-obstructed kidney was only observed three days following the creation of the UUO but disappeared six days following the reversal of the UUO. Arabic gum ameliorated this early rise in MDA level. Likewise, AG significantly attenuated the rise in the antioxidant SOD in the left obstructed kidney when measured three days following the creation of the UUO. However, AG did not affect the level of GSH in the obstructed kidney. This is probably due to the fact that the tissue level of GSH was similar in both the obstructed and control kidneys. Glutathione (GSH) is a free radical scavenger and the lack of change in the level of GSH as a result of UUO could possibly be due to the consumption of GSH during the breakdown of free radicals. Similar findings were observed in other tissues by other researchers [30]. Collectively, these results confirm the antioxidant protective effect of AG, which has been reported previously in other renal conditions, such as adenine-induced chronic kidney disease [22].

In addition to attenuating the changes in some of the oxidative stress markers, AG attenuated the UO-induced early rise in the TNF-α gene expression. TNF-α was shown to be one of the acute inflammatory mediators in UO [31,32,33]. For example, it has been demonstrated that both the serum [34] and tissue mRNA levels of TNF-α [35] increase sharply soon after UO, which is consistent with the findings of this study in which the rise in gene expression of TNF-α and other cytokines was observed three days after the creation of the obstruction but had returned to normal six days after the release of the UUO. In the kidney, TNF-α is produced by either kidney tissues, such as mesangial or tubular cells [36,37], or by the infiltrating inflammatory cells [38]. Regardless of the exact source of TNF-α in this model of UUO, the attenuation of the gene expression in the treated group indicated a potential protective effect of AG in this condition.

Similar to TNF-α, TGF-β1, which is a potent pro-fibrotic and pro-apoptotic cytokine, was proved to be involved in renal disease [39,40]. Renal TGF-β1 mRNA expression has been shown to increase considerably after the onset of obstruction [41] and plasma level of TGF-β1 increases in patients with ureteric calculi [42].

In addition to its effect on TNF-α and TGF-β1, AG decreased the gene expression of p53, the renal expression of which has been demonstrated to increase following UO [43]. p53 has traditionally been known for its proapoptotic properties. However, without assessment of apoptosis, one cannot determine with certainty if the significant difference in p53 gene expression observed between the treated and untreated groups was associated with a significant effect on apoptosis. Further, p53 has other functions apart from being proapoptotic, such as its role in autophagy [44]. Hence, this significant difference in p53 gene expression could be associated with apoptosis, autophagy, or other unknown functions of P53, and further tests are required to clarify this issue. Regardless of the exact role of a p53 rise in UO, the attenuation in the gene expression of TNF-α, TGF-β1, and p53 in the current study indicates a protective effect on the UO-induced inflammation and on the on-going renal fibrosis observed in obstructive nephropathy.

The effect of AG on pro-inflammatory cytokines and oxidative stress markers was associated with significant histological protective features. The degree of tubular dilatation in the AG-treated group three days after the creation of the UUO was significantly less than in the control group. The degree of tubular dilatation following ureteric obstruction depends on several factors, including the degree of ureteric obstruction, glomerular filtration rate, and the integrity of the tubular wall. In the current study, the effect of AG on the extent of tubular dilatation is unlikely to be due to the first two factors, as both groups had complete ureteric obstruction for three days. Further, the results showed a similar GFR in the left kidney in both Vx-2 and AG-2 groups. Hence, the likely cause for the effect of AG on tubular dilatation was its effect on tubular wall integrity due to its anti-inflammatory and antioxidant effects, as shown by the current data. The lack of any significant difference in the inflammatory infiltration between the AG-treated groups and the control group is difficult to ascertain but is probably due to fact that the inflammatory reaction was too mild to enable detection of any differences despite the significant difference between the gene expression of proinflammatory cytokines. This protective effect of AG on tubular histology was associated with a tendency to have a protective effect on the UO-induced renal tubular dysfunction, as shown by the improvement in the fractional excretion of sodium in the post-obstructed kidney, although this did not reach statistical significance. This effect is similar to the findings of other studies in other renal pathological conditions. For instance, one- or two-week treatment with AG has been reported to improve the fractional excretion of sodium in diabetic mice [45].

In the current study, despite the anti-inflammatory, antioxidant, and histological protective effects of AG, it did not affect the renal hemodynamic parameters. The cause of this is difficult to ascertain from the current data but could possibly be due to the lack of a strong effect of AG on one or more of the major systems that control the hemodynamics of the kidney and leads to the alterations observed following UO, such as the renin–angiotensin system, prostaglandins, and endothelins [2,4,9,10]. Similarly, AG did not affect the blood pressure in the treated group. In some previous work, AG was shown to decrease the high blood pressure in animals with chronic kidney disease [46]. Others, however, failed to show such an effect in a similar model [47]. In diabetic mice, AG ameliorated the associated rise in blood pressure [45]. Regardless of the reason for this controversy, none of the previous work has shown an effect of AG in normotensive animals similar to findings in this study.

From the current study, it is difficult to ascertain which particular ingredient of AG, which is a branched-chain, complex polysaccharide, has the protective effect seen in this model. Indeed, studies have shown that the composition and chemical properties of AG might vary according to species and other factors related to the origin of the gum [17]. Therefore, further research is required to elucidate which polysaccharide or salt has these effects.

One of the limitations of this study is the lack of measurement of the level of pro-inflammatory and profibrotic markers at the protein level in addition to the measurement at the mRNA level. In view of this and the lack of any significant difference in the inflammatory reaction between the treated and untreated groups, regardless of the reason (*vide supra*), it might be concluded that AG has a more significant effect on the oxidative stress markers in this condition at the time points used in this experiment.

This model of a reversible UO was selected due to its similarity to the clinical scenario of a ureteric stone during its passage from the kidney to the urinary bladder causing a transient obstruction to the kidney. The validity of the model was demonstrated by the significant drop in the GFR and RBF in the obstructed kidney even six days after the reversal of the UO, similar to what was reported in other studies [6,48]. The beneficial effect of AG on the kidney might be of clinical interest in patients with obstructing ureteric calculus, especially those with a single kidney or those with compromised renal functions.

## 5. Conclusions

The administration of Arabic gum before and during relatively long period of ureteric obstruction appears to have attenuated the UUO-induced alterations in oxidative stress markers, gene expression of some of the inflammatory and fibrotic cytokines, and in histological features. This was associated with a tendency to improve the renal tubular functional parameters when measured six days following the reversal of obstruction. These protective effects might have useful implications in a clinical setting.

## Figures and Tables

**Table 1:** Forward and reverse primers and fluorogenic probe sequences used for real time quantitative PCR analysis.

Gene	Gene Bank Reference		5′–3′ Sequence
TNF-α	NM_012675.3	Forward	GGCTCCCTCTCATCAGTTCCAT
		Reverse	CGCTTGGTGGTTTGCTACG
		Probe	dFAM-CCCAGACCCTCACACTCAGATCATC-BHQ-1
TGF-β1	NM_021578.2	Forward	GTGGCTGAACCAAGGAGACG
		Reverse	CGTGGAGTACATTATCTTTGCTGTC
		Probe	dFAM-ACAGGGCTTTCGCTTCAGTGCTC-BHQ-1
p53	NM_030989.3	Forward	CGAGATGTTCCGAGAGCTGAATG
		Reverse	GTCTTCGGGTAGCTGGAGTG
		Probe	dFAM-CCTTGGAATTAAAGGATGCCCGTGC-BHQ-1
